# ﻿A new species of *Liparis* (Orchidaceae, Epidendroideae, Malaxidinae) from the Bosque de Protección Alto Mayo, San Martín, Peru

**DOI:** 10.3897/phytokeys.224.98654

**Published:** 2023-04-07

**Authors:** José D. Edquén, Jessy P. Arista, Alexander Damián, Gerardo A. Salazar

**Affiliations:** 1 Facultad de Estudios de Posgrado, Universidad Nacional Toribio Rodríguez de Mendoza de Amazonas, Chachapoyas, Amazonas, Peru; 2 Herbario KUELAP, Facultad de Ingeniería y Ciencias Agrarias, Universidad Nacional Toribio Rodríguez de Mendoza de Amazonas, Chachapoyas, Amazonas, Peru; 3 Department of Botany, University of Wisconsin-Madison, Madison, Wisconsin 53706, USA; 4 Departamento de Botánica, Instituto de Biología, Universidad Nacional Autónoma de México, Mexico City, Mexico

**Keywords:** Andean cloud forest, high fruit set, labellum, self-pollination, Alta producción de frutos, autopolinización, bosque nublado andino, labelo

## Abstract

*Liparisaltomayoënsis***sp. nov.** is described, illustrated, and tentatively assigned to the Neotropical section Decumbentes on the basis of its branching, prostrate rhizomes and upright stems bearing several leaves. Vegetatively, the new species is distinguished by its short, upward stems bearing 3–6 leaves, these with undulate, translucent margins and reticulate, prominent veining on the upper surface. Florally, it is distinctive in the labellum with fleshy basal one-half provided with a central, rounded cavity limited on each side by a prominent, bilobulate ridge and apically by a lunate ridge, and membranaceous, trilobulate apical one-half deflexed ca. 90°. In contrast with other species of section Decumbentes, in which fruit formation is infrequent, in *L.altomayoënsis* a high proportion (⁓50–100%) of flowers develop into a fruit; in some flowers the pollinaria rotate and contact the stigma, apparently resulting in at least facultative self-pollination. The main differences among the six species of L.sectionDecumbentes hitherto known are contrasted in a dichotomous key. The new species is known only from three populations located in the Bosque de Protección Alto Mayo, on the Amazonian slope of the Andes in northeastern Peru but appears to be under no foreseeable threats.

## Abstract (es)

*Liparisaltomayoënsis***sp. nov.** es descrita, ilustrada y asignada tentativamente a la sección neotropical *Decumbentes* con base en sus rizomas postrados, ramificados y tallos ascendentes portando varias hojas. Vegetativamente, la nueva especie se distingue por sus tallos ascendentes cortos con 3–6 hojas, éstas con márgenes undulados, translúcidos y venación reticulada prominente en la superficie superior. Floralmente, es distintivo su labelo con la mitad basal carnosa, provista de una cavidad central redondeada que está limitada a cada lado por una cresta bilobulada prominente y apicalmente por una cresta lunada, y la mitad apical del labelo membranácea, trilobulada y deflexa ca. 90°. En contraste con otras especies de la sección *Decumbentes*, en las que la producción de frutos es infrecuente, en *L.altomayoënsis* una *alta* proporción (⁓50–100%) de las flores produce fruto; en algunas flores los polinarios rotan y contactan el estigma, aparentemente resultando en autopolinización al menos facultativa. Las diferencias principales entre las seis especies de *L.* sección *Decumbentes* conocidas hasta ahora se contrastan en una clave dicotómica. La nueva especie se conoce solamente de tres poblaciones localizadas en el Bosque de Protección Alto Mayo, en la vertiente amazónica de los Andes en el noreste de Perú, pero no parece estar sujeta a amenazas previsibles.

## ﻿Introduction

The genus *Liparis* Rich. consists of about 320 epiphytic and geophytic species and is widely distributed in tropical, subtropical, and temperate regions of the Old and New Worlds, being distinguished from other members of Malaxidinae mainly by the elongate column with an apical, incumbent anther (Ridley 1886; Cribb et al. 2005). Although several molecular phylogenetic studies have shown that *Liparis* is polyphyletic (Cameron 2005; Tang et al. 2015; Li et al. 2020; Ya et al. 2021; Wang et al. 2022), such studies have been strongly biased towards tropical/subtropical Asian taxa. Many Neotropical species have not yet been included in molecular analyses, and much work remains to be done to attain a clear picture of generic limits and relationships in this region. Meanwhile, the morphology-based sectional classification proposed by Garay and Romero-González (1999) provides a framework for taxonomic discussion and comparison of morphologically discrete groups.

**Figure 1.**
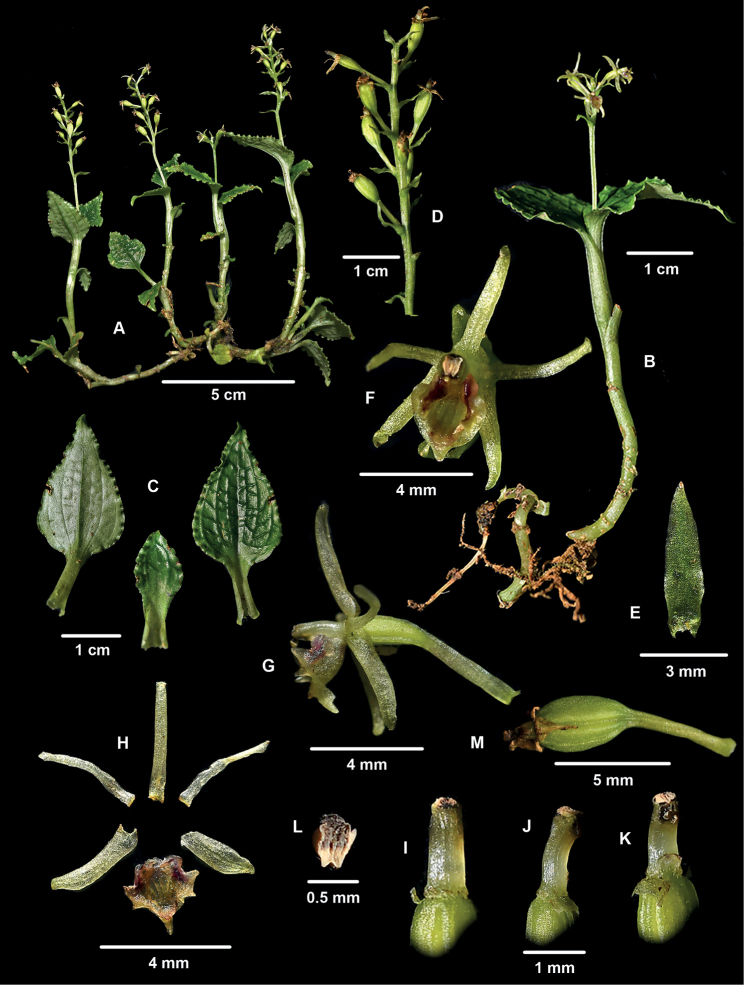
*Liparisaltomayoënsis* (from *Edquén 6111*) **A** habit **B** flowering stem **C** leaves **D** infructescence **E** floral bract **F** flower from front **G** flower from side **H** perianth dissection **I** column, dorsal view **J** column, lateral view **K** column, ventral view **L** anther **M** developing capsule.

LiparissectionDecumbentes Garay & G.A.Romero is endemic to the Neotropics and distinctive in the decumbent, creeping stems with distichously arranged leaves (Garay and Romero-González 1999; Fig. 1). This poorly known group is restricted to Andean cloud forests from Venezuela south to Bolivia, and included five species, two of them only recently described from Peru (Damián et al. 2020; Salazar et al. 2022).

In this work, we propose an additional species tentatively assignable to LiparissectionDecumbentes, discovered during fieldwork conducted as part of our ongoing orchid inventory of the Bosque de Protección Alto Mayo, San Martín, Peru (BPAM; J. D. Edquén et al. in prep.). The new species is described and illustrated, and the features permitting to distinguish the currently known six members of LiparissectionDecumbentes are compared in a dichotomous key.

## ﻿Materials and methods

Live plants were studied *in situ* and photographed with a digital camera (Nikon 850, Nikon Corporation, Tokyo, Japan) provided with a 60 mm AF Micro Nikkor lens (Nikon). Leaves and flowers preserved in ethanol 70% were examined and photographed under a stereomicroscope (Stemi SV 6, Carl Zeiss Mikroskopie, Jena, Germany) using a cell phone (iPhone 11, Apple Inc., Cupertino, USA). All images were processed for plate preparation with ADOBE PHOTOSHOP v. 24.0.1 (Adobe Inc., San Jose, USA). Three specimens from different locales were pressed and deposited in the herbarium of the Universidad Nacional Toribio Rodríguez de Mendoza, Chachapoyas, Peru (KUELAP); one of them was designated as the holotype. Measurements were made on the pressed specimen and the alcohol-preserved specimens. Our material was compared with the protologues and additional literature, types, and records of types of all previously described species of New World *Liparis*, especially those belonging to section Decumbentes, to which our material shows similarities. The collections of several major herbaria in Peru and abroad were studied, including AMES, AMO, CUZ, F, GH, HOXA, K, KUELAP, MEXU, MO, MOL, NY, QCE, QCNE, UFV, US, and USM (herbarium acronyms according to Thiers 2022). The new species was compared on morphological grounds to other members of section Decumbentes and the main differences were incorporated into a key to the six hitherto recognized species belonging to this section.

## ﻿Taxonomic treatment

### ﻿ Liparis altomayoënsis

Taxon classificationPlantaeScorpaeniformesLiparidae

Salazar & Edquén sp. nov.

6D5D530B-0E70-59CA-B263-24601B812BCE

urn:lsid:ipni.org:names:77317365-1

Figs 1 , 2

#### Type.

Peru. Departamento San Martín: Provincia Rioja, distrito Pardo Miguel Naranjos, sector Venceremos, camino al terreno del Sr. Roner Espinal Gómez, 5°41'10.68"S, 77°45'19.17"W, 1756 m a.s.l., 15 June 2022, *J. D. Edquén 6111* (holotype: KUELAP 002579!).

#### Diagnosis.

**Figure 2.**
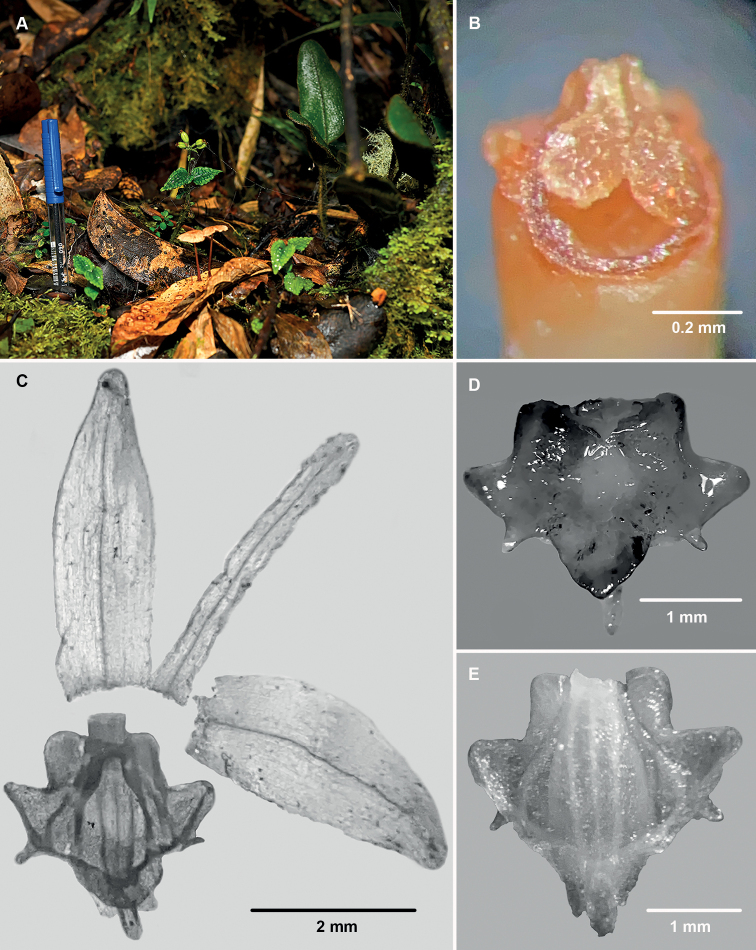
*Liparisaltomayoënsis* (from *Edquén 6111*) **A** plants in habitat (the pen serves as size reference) **B** column apex from below showing the two pollinaria on the stigmatic cavity **C** dissection of the perianth **D** labellum, ventral view **E** labellum, dorsal view.

*Liparisaltomayoënsis* is characterized by the short prostrate rhizomes and upright stems (to 5 and 8 cm long, respectively); 3–6 spirally arranged leaves per stem; leaves petiolate, the blades with strongly undulate, translucent margins and reticulate veining prominent on the upper surface and sunken on the underside. The labellum is slightly wider than long, its base provided at each side with a fleshy, rounded, channeled, erect lobule forming a tunnel with the lower half of the column; basal one-half of labellum provided with a central, rounded cavity limited on each side by a prominent, bilobulate ridge and apically by a lunate ridge; apical one-half of labellum membranaceous, trilobulate, deflexed ca. 90°. (Figs 1F–H, 2C–E).

#### Description.

Terrestrial, decumbent, glabrous ***herb*** 5–15 cm tall including the inflorescence. ***Roots*** scarce, dull white, glabrous, arising from the rhizome, up to 15 mm long, ca. 0.5 mm in diameter. ***Rhizome*** (prostrate portion of the stems) branching, terete, greenish white, each branch formed by several (up to 10) internodes, 2–5 cm long, 2–3 mm in diameter, partially covered by brownish remains of cataphylls; upright portion of the stem 3.5–8 cm long, 2.5–3.5 mm in diameter, formed by 4–6 internodes, these nearly completely covered by the leaf sheaths. ***Leaves*** [2–]3–6 per stem, arranged into a spiral, petiolate; petiole (4–)10–14 × 3–5 mm, semi-tubular, obliquely sheathing the internode; blade 10–25 × 7–15 mm, ovate, acute to shortly acuminate, margins strongly undulate, translucent, 5–7 main parallel veins and several transverse ones, all veins conspicuously raised on the upper surface and slightly sunken on the underside, excepting the slightly prominent central vein; upper surface glossy dark green, lower surface opaque olive green. ***Inflorescence*** terminal, 4–7 cm long; peduncle 20–26 mm long, 1–1.5 mm in diameter, with several longitudinal low keels; raceme 2–6 cm long, moderately lax, with 7–20 flowers opening in succession, but most can be open at a time. ***Floral bracts*** shorter than the ovaries, divergent from the rachis at flowering, patent at fruiting stage, pale green, lanceolate, acute, 5–7 × 1.3–1.5 mm. ***Ovary*** spreading, pedicellate, narrowly obconical, convex dorsally, flat ventrally, slightly 3-angled, 6–6.6 mm long, 1–1.2 mm wide above the middle; about one half of the length corresponds to the twisted pedicel. ***Flowers*** resupinate, pale green with a wine–colored ridge at each side of the central cavity of the labellum. ***Sepals*** spreading, with revolute margins, 1-veined; lateral sepals obliquely elliptic, rounded, 3.6–3.7 × 1.6–1.7 mm, dorsal sepal linear–lanceolate, rounded and slightly calyptrate at apex, 4–4.1 × 1.2–1.3 mm. ***Petals*** spreading, incurved, linear, slightly falcate, rounded, 4–4.2 × 0.5–0.6 mm. ***Labellum*** 2.6–2.7 mm total length, 2.7–2.8 mm total width when spread out, sessile, 7-veined, in natural position its basal one-half diverging ca. 60° from the column and the apical one-half in turn deflexed ca. 90°; base provided at each side with a fleshy, rounded, channeled, erect lobule forming a tunnel with the lower half of the column; disc fleshy, deeply concave, provided at each side of the cavity with a obliquely triangular, retrorse, rounded lobe ca. 1 × 0.8 mm, which has an erect ridge projected towards the apex into an acute, narrowly triangular lobule ca. 0.2 × 0.1 mm; cavity limited apically by a transverse, lunate, rounded to obtuse fleshy ridge; apex membranaceous, trilobulate, the lobules rounded, mid-lobule ca. 0.3 × 0.2 mm, lateral lobules much shorter, deflexed in natural position. ***Column*** semiterete, clavate, slightly arcuate, lacking auricles, whitish green below the middle, dark green with purplish suffusion near the apex, 1.6–1.8 × 0.7–0.8 mm. ***Anther*** apical, incumbent, transverse to the main column axis, whitish, cordiform, emarginate, 2-celled with each cavity partially subdivided in two, ca. 0.2 × 0.4 mm. ***Pollinaria*** 2, each consisting of 2 fused pollinia, yellow, obliquely ovoid, granulose, 0.3–0.4 × ca. 0.2 mm. ***Capsule*** ascending, ellipsoid, with 6 low longitudinal ribs, to 5 × 3.5 mm plus a filiform pedicel ca. 4.5 mm long, when mature yellowish brown.

#### Phenology.

Flowering recorded in June and July. Capsules in different stages of development were observed from June to October. Mature, empty dehiscent capsules from the previous year’s flowering were observed in mid-May.

#### Distribution and habitat.

Known only from sector Venceremos of the BPAM. Terrestrial, in deep leaf mold on steep slopes with wet montane cloud forest on a steep tepui (table mountain) slope dominated by dwarfed trees of *Clusia*L. (Clusiaceae), *Meriania* Sw., *Miconia* Mart. (Melastomataceae) and stands of *Chusquea* Kunth (Poaceae), at 1750–2160 m a.s.l.

#### Etymology.

The specific epithet refers to the Bosque de Protección Alto Mayo, the protected natural area in northeastern Peru where this species was discovered.

#### Taxonomic notes.

We tentatively include the new species in LiparissectionDecumbentes because of its branching, prostrate rhizomes and upright stems bearing several leaves (Fig. 1A, B). However, in many other respects it differs from the five previously known species of the section, and its systematic position will have to be revised when material suitable for molecular analysis is available. Vegetatively, *L.altomayoënsis* differs from all other species of section Decumbentes in its comparatively short, upward stems bearing only a few (3–6) spirally arranged leaves with strongly undulate, translucent margins and reticulate veining, with the veins prominent on the upper surface and sunken on the underside (Fig. 1C). Florally, the most distinguishing feature of the new species is the unusual morphology of the labellum, which is slightly wider than long. The basal one-half of the labellum is fleshy, diverges from the column about 60° and has a retrose lobe on each side and a central, rounded cavity limited on each side and the apex by prominent ridges; the apical one-half of the labellum is membranaceous, deflexed ca. 90° with respect to the basal one-half, and 3-lobulate (Fig. 1F, G). The lateral labellum ridges consist of a proximal, retrorse, obtuse lobule and a forwardly projecting, narrowly triangular distal lobule. The apical ridge limiting the cavity is unlobed, lunate, and rounded or obtuse. The column is semiterete, clavate, slightly arcuate, lacking auricles and the anther is terminal, transverse to the main axis of the column (Fig. 1E, I–K). The features allowing for the distinction of the six species hitherto known of L.sectionDecumbentes are highlighted in the key (see below).

#### Reproductive biology.

Unlike other species of LiparissectionDecumbentes, in which fruit production seems to be very rare (cf. Damián et al. 2020; Salazar et al. 2022), a surprisingly high percentage (⁓50–100%) of flowers of the plants of *L.altomayoënsis* we examined were developing into a fruit (Figs 1A, B, 2A). Such high frequency of fruit formation is similar to that recorded in self-pollinating populations of other, distantly related species of *Liparis*, such as eastern Asian *L.kumokiri* F.Maek. of section Liparis (Oh et al. 2001). We were unable to verify in the field possible evidence of self-pollination, but we could not remove the pollinaria of several fresh flowers examined and photographed *in situ*, and subsequent examination of the columns of six alcohol-preserved flowers under a stereomicroscope revealed that, in two of them, the two pollinaria were in contact with the stigmatic cavity, as if they had rotated downwards with the rostellum acting as a sort of hinge (Fig. 2B). A similar rotation of the pollinaria to contact the stigma has been suggested as a mechanism of self-pollination, probably promoted by the dislodgement of the anther by raindrops, in other species of *Liparis* such as *L.loeselii* (L.) Rich. in eastern North America (Catling 1980) and *L.kumokiri* in Japan (Suetsugu 2019). Facultative autonomous self-pollination resulting from rotation of the pollinarium such that the pollinia contact the stigma has been recorded in some populations of species of other Epidendroideae genera, such as *Eulophiaalta* (L.) Fawc. & Rendle (Goss 1973; G.A. Salazar, pers. obs.), *Eulophiamaculata* (Lindl.) Rchb.f. (as *Oeceocladesmaculata* (Lindl.) Lindl.; Aguiar et al. 2012: fig. 4), and various species of *Corallorhiza* Gagn. (Catling 1990 and references therein; Freudenstein 1997; G. A. Salazar pers. obs.). Hence, there is a possibility that at least some of the many capsules observed in *L.altomayoënsis* may have resulted from self-pollination by the spontaneous rotation of the pollinaria. However, in fresh flowers of *L.altomayoënsis* the labellum is distinctive glossy, especially the raised borders of the basal cavity and the bottom of the cavity itself, suggesting nectar mimicking, as proposed for other *Liparis* having a glossy central band along the labellum (Oh et al. 2001). We were unable to verify whether the cavity contains nectar, which has been shown to be present at least in small quantities in some species of *Liparis* (Margońska et al. 2019; Suetsugu 2019). The presence of nectar or a nectar-mimicking glossy surface are suggestive of visitation and probable cross-pollination mediated by insects. At the present time, it is not clear whether the high fruit set observed in *L.altomayoënsis* is the result of self-pollination, pollinator-mediated cross pollination, or both, and the factors underlying its high success in setting fruit will have to be clarified by carefully designed field and laboratory experiments.

#### Conservation assessment.

The BPAM was established in 1987 by the Peruvian government to protect the water sources for agriculture, industrial use, and human consumption in the valley of the Upper Mayo River, as well as to conserve the fauna and flora (Servicio Nacional de Áreas Naturales Protegidas por el Estado 2023). It encompasses 182,000 ha of rugged mountainous terrain on the eastern (Amazonian) slope of the Andes in the northwestern portion of the Department San Martín and adjacent areas of Departments Amazonas and Loreto (ca. 5.4°–6.2°S, 77.2–77.8°W), covering an elevation interval from ca. 900 to 3800 m a.s.l. The vegetation includes wet lower montane forest, montane rain/cloud forest, and high-elevation grassland. *Liparisaltomayoënsis* is known only from three stands (populations) of various dozen plants located on the northwestern portion of the BPAM (sector Venceremos) on a steep tepui slope. There were no signs of human alteration or potential risk factors to the populations, which are under legal protection within the BPAM. Moreover, there are large expanses of potentially suitable habitat that remain to be explored, which suggests that this species is not an immediate conservation concern, as long as its habitat remains unaltered.

#### Additional specimens examined.

Peru. As the type locality, 5°42'41.55"S, 77°44'19.54"W, 2090 m a.s.l., 17 May 2022, *J. D. Edquén 6101* (KUELAP!); as the type locality, 5°42'42.73"S, 77°44'31.99"W, 2160 m a.s.l., 4 July 2022, *J. D. Edquén 6421* (KUELAP!).

### ﻿Key to the species of LiparissectionDecumbentes

**Table:** 

1	Labellum saddle-shaped, i.e., strongly convex with the lateral margins downcurved, distal margins laciniate, apex projected into a narrowly triangular lobule	***L.inaudita* Salazar, Edquén & D.Trujillo**
–	Labellum not saddle-shaped, at most slightly convex without downcurved margins, concave or strongly revolute, distal margins entire or erose, apex rounded, shallowly emarginate, apiculate, mucronate or 3-lobulate, if prominently apiculate then the margins entire (not laciniate)	**2**
2	Labellum strongly revolute, when spread out abruptly expanded from a short cuneate base, about two times wider than long, transversely oblong–flabellate, apex apiculate	***L.laticuneata* C.Schweinf.**
–	Labellum not strongly revolute, when spread out variously shaped but never abruptly expanded from a short cuneate base, longer than wide or only slightly wider than long, apex shallowly emarginate with a small apicule in the sinus or 3-lobulate	**3**
3	Labellum deeply concave, the concavity limited at each side and towards the apex by prominent, fleshy ridges, lateral ridges retrorse, projected forwardly into a narrowly triangular lobule, apical ridge lunate, obtuse, labellum apex 3-lobulate	***L.altomayoënsis* Salazar & Edquén**
–	Labellum flat, slightly convex or slightly concave, without ridges whatsoever, labellum apex not 3-lobulate	**4**
4	Leaves sessile; labellum slightly convex, when spread out ovate–elliptic, apex and base rounded	***L.sessilis* Damián, Salazar & Rimarachín**
–	Leaves petiolate; labellum flat or slightly concave, when spread out obovate, pandurate, or ovate–rhombic, apex obtuse or shallowly emarginate, mucronate, base cordate	**5**
5	Flowers with pale green sepals and petals, and red purple labellum; labellum ovate–rhombic, obtuse; column slender above a thick base, strongly arcuate, about four times as long as wide or longer	***L.crispifolia* Rchb.f.**
–	Flowers entirely green with a darker green central stripe on the labellum; labellum obovate or pandurate, shallowly emarginate, the sinus apiculate; column thick throughout, slightly arcuate, about 2.5 times longer than wide	***L.brachystalix* Rchb.f.**

## Supplementary Material

XML Treatment for Liparis altomayoënsis
